# Activation of AKT pathway by Nrf2/PDGFA feedback loop contributes to HCC progression

**DOI:** 10.18632/oncotarget.11700

**Published:** 2016-08-30

**Authors:** Danyang Liu, Yonglong Zhang, Yingze Wei, Guoyuan Liu, Yufeng Liu, Qiongmei Gao, Liping Zou, Wenjiao Zeng, Nong Zhang

**Affiliations:** ^1^ Department of Pathology, School of Basic Medical Sciences, Fudan University, Shanghai, China; ^2^ Department of Biliary-Pancreatic Surgery, Renji Hospital, Shanghai Jiao Tong University, Shanghai, China; ^3^ Department of Pathology, Tumor Hospital of Nantong, Nantong, China; ^4^ Department of Pathology, Huashan Hospital, Fudan University, Shanghai, China

**Keywords:** Nrf2, PDGFA, hepatocellular carcinoma, AKT, tumorigenesis

## Abstract

Nuclear factor erythroid-2-related factor 2 (Nrf2), a master transcription factor in the antioxidant response, has been found to be ubiquitously expressed in various cancer cells and in the regulation tumor proliferation, invasion, and chemoresistance activities. The regulatory roles of Nrf2 in controlling Hepatocellular carcinoma (HCC) progression remain unclear. In this study, we demonstrated that Nrf2 was significantly elevated in HCC cells and tissues and was correlated with poor prognosis of HCCs. Consistently, Nrf2 significantly promoted HCC cell growth both *in vitro* and *in vivo.* Further investigation suggested a novel association of Nrf2 with Platelet-Derived Growth Factor-A (PDGFA). Nrf2 promoted PDGFA transcription by recruiting specificity protein 1 (Sp1) to its promoter, resulting in increased activation of the AKT/p21 pathway and cell cycle progression of HCC cells. As a feedback loop, PDGFA enhanced Nrf2 expression and activation in an AKT dependent manner. In line with these findings, expression of Nrf2 and PDGFA were positively correlated in HCC tissues. Taken together, this study uncovers a novel mechanism of the Nrf2/PDGFA regulatory loop that is crucial for AKT-dependent HCC progression, and thereby provides potential targets for HCC therapy.

## INTRODUCTION

Hepatocellular carcinoma (HCC) is one of the most prevalent cancers worldwide, ranking as the third leading cause of cancer-related death [1]. Emerging evidence has established that understanding the molecular landscape of HCC progression will contribute to diagnosis, prevention and potentially even treatment [2]. Nuclear factor erythroid-2-related factor 2 (Nrf2) is a master regulatory transcription factor that activates transcriptional programs in response to oxidative stress, inflammation, apoptosis and metabolism [3, 4]. Nrf2 activity is negatively regulated by Kelchlike ECH-associated protein 1 (KEAP1), which is an adaptor protein for ubiquitination and subsequent degradation of Nrf2 under quiescent conditions [5]. In the presence of high ROS levels, Nrf2 is liberated from KEAP1 and translocates to the nucleus, where it transactivates ARE driven gene expression with other bZIP proteins such as small musculoaponeurotic fibrosarcoma (Maf) proteins [6].

Nrf2 has been reported to be abundantly expressed and regulate proliferation, invasion, and chemoresistance activities in various cancer cells [7–9]. In HCC, Nrf2 plays protective roles in hepatic inflammation, fibrosis, and hepatocarcinogenesis [10, 11]. Inhibition of Nrf2 expression and activity *in vitro* and *in vivo* increased the anticancer activity of erastin and sorafenib in HCC cells [12]. Nrf2/KEAP1 mutations are present in most early and advanced HCCs and functional experiments demonstrate that Nrf2 is an oncogene critical for HCC progression and development [10]. However, the way in which Nrf2 promotes HCC progression remains poorly understood.

PDGFA (Platelet-Derived Growth Factor-A) has long been associated with poor prognosis and high metastatic rate [13]. Interaction of PDGFA with its receptor leads to cellular responses such as proliferation and migration through PI3K/AKT and MEK signaling [14, 15]. *In vivo*, PDGF plays a significant role in angiogenesis, proliferation, and metastasis [16]. The PDGF family consists of 5 isoforms that exert their cellular effects by binding to their receptors PDGFRα or PDGFRβ with different affinities. PDGFA binds to PDGFRα, inducing PDGFR phosphorylation and activating their downstream pathways involved in several oncogenic mechanisms. Indeed, inhibition of PDGFA signaling has been shown to reduce growth and metastasis of human HCCs [17, 18]. Nrf2 and the PDGFA/AKT signaling pathway are both dysregulated in HCC tumorigenesis, however, the relationship between them has not been fully explored.

In this study, we find Nrf2 serves as a novel regulator of PDGFA that significantly transactivates its mRNA transcripts, which subsequently activates the AKT/p21 pathway, resulting in increased cell cycle progression of HCC cells. As a feedback loop, PDGFA up-regulates Nrf2 expression and promotes Nrf2 nuclear translocation and target gene expression. Therefore, our studies propose a novel mechanism whereby the Nrf2/PDGFA feedback loop might play a critical role in HCC progression.

## RESULTS

### Nrf2 is up-regulated in HCC tissues and is associated with poor prognosis

**Figure 1:**
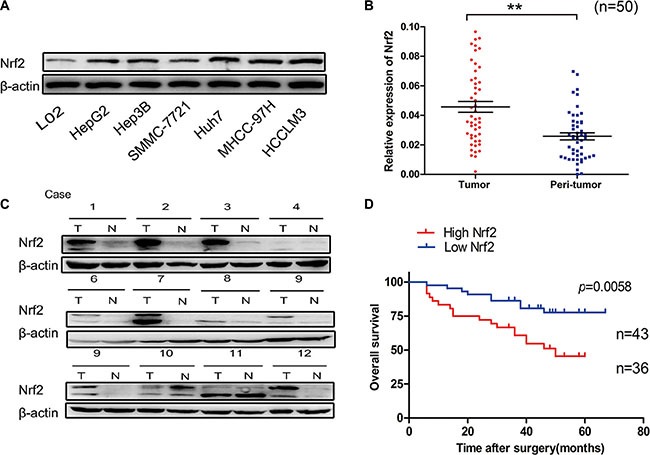
Nrf2 is significantly up-regulated in HCC (**A**) Expression of Nrf2 was higher in a panel of HCC cell lines. The expression of Nrf2 in indicated HCC cell lines was subjected to western blot analysis using anti-Nrf2 antibody. (**B**) The mRNA levels of Nrf2 were elevated in HCC tissues. The mRNA transcripts of Nrf2 were measured by qRT-PCR using specific primers of Nrf2 in HCC tissues and normal counterparts. (**C**) The protein levels of Nrf2 were up-regulated in HCC tissues. The whole cell lysates of HCC tissues and normal counterparts were extracted and measured by western blot analysis using anti-Nrf2 antibody. (**D**) Overall survival (OS) differences between patients with high and low levels of Nrf2 protein expression. IHC results were independently graded by two experienced pathologists. Tissue samples with grades 0 (no staining) and 1(weak staining) were grouped as ‘low’ expression, and samples with grade 2 (strong staining) were grouped as ‘high’ expression. Survival curve was generated by using Graphpad software Prism 5 and Log Rank Mantel-Cox Test analysis. ^*^P < 0.05; ^**^P < 0.01. Student's-*t* test was used for the statistical analysis.

To explore the mechanisms underlying Nrf2 regulated HCC progression, we first determined the expression and significance of Nrf2 in HCC cell lines and tissues. The protein level of Nrf2 in a panel of HCC cell lines was higher in comparison to L02, a normal hepatocyte cell line (Figure 1A). In accordance with the results in cell lines, the protein levels and mRNA transcripts of Nrf2 were markedly up-regulated in HCC tissues in contrast to adjacent counterparts, implying oncogenic activity in HCCs (Figure 1B and 1C). Moreover, survival analysis showed that patients with high levels of Nrf2 in HCC tissues had a poorer prognosis than those with low levels of Nrf2 (Log Rank (LR) = 4.787, *p* = 0.0287) (Figure 1D).

### Nrf2 promotes HCC cell proliferation *in vitro* and *in vivo* by up-regulating cell cycle progression

**Figure 2:**
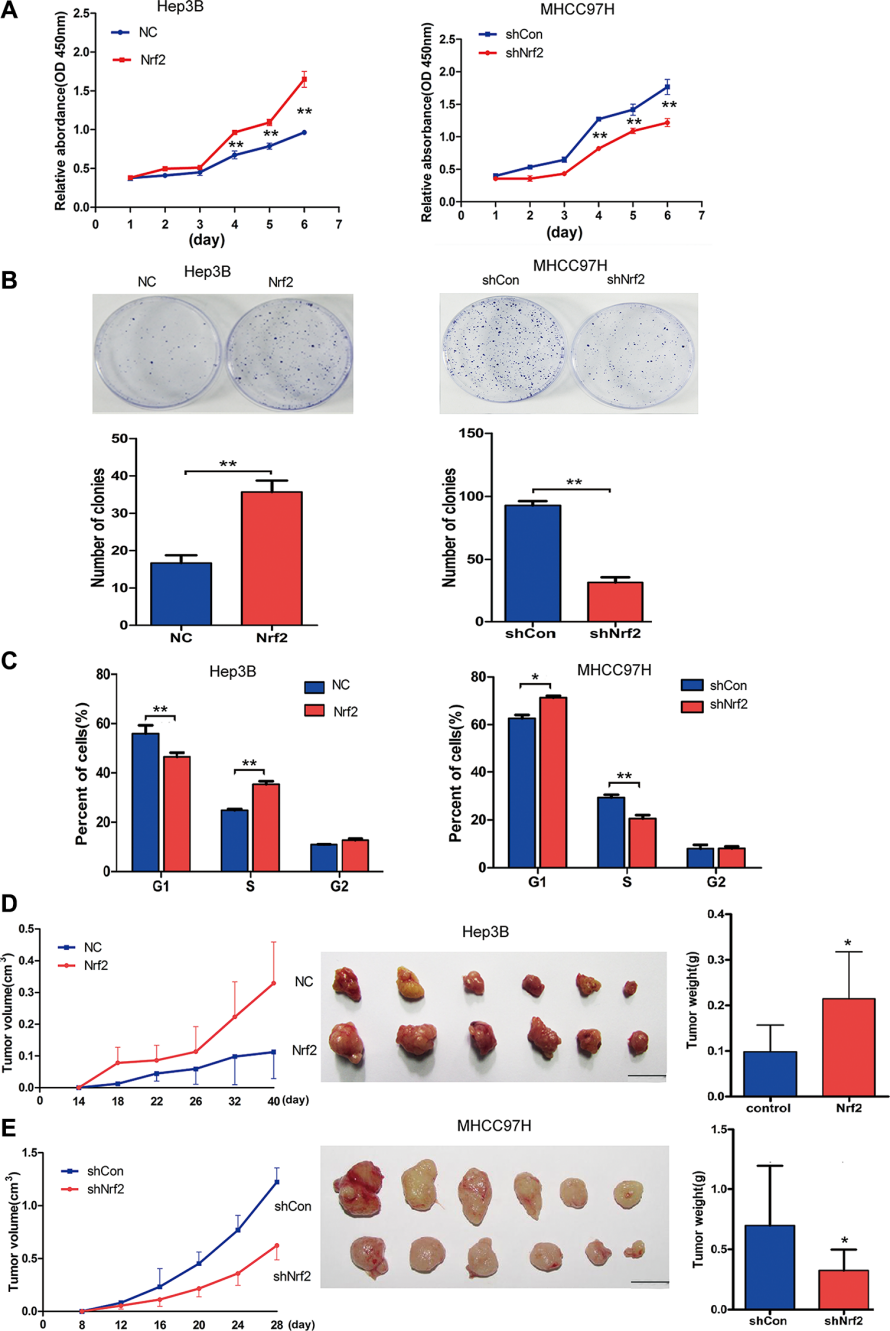
Nrf2 promotes HCC cell proliferation by up-regulating cell cycle progression both *in vitro* and *in vivo* (**A**) Nrf2 triggered HCC cell growth in Hep3B and MHCC-97H cells. Equal numbers of the cells infected with indicated lentivirus were plated into 96-well plates. The proliferation activity was measured by CCK8 assay. (**B**) Nrf2 promoted the colony formation ability of HCC cells. 500–1000 cells that infected with indicated lentivirus was plated into 35 cm dish for 2 weeks. The number of colonies was counted and calculated. (**C**) Nrf2 controlled HCC cell cycle progression. 10^6^ cells that infected with indicated lentivirus were analysed by FACS for the relative percentages of cell cycle phases. Means ± SD from three independent experiments are presented. ^*^P < 0.05; ^**^P < 0.01. Student's-*t* test was used for the statistical analysis. (**D**) Ectopic expression of Nrf2 promoted tumor growth *in vivo*. Left, growth curve of tumor volumes at indicated time. Middle, demonstration of tumor volumes of xenograft tumors removed from indicated group. Right, Statistical analysis of tumor weight of the resected tumors. (**E**) Depletion of Nrf2 repressed tumor growth *in vivo*. Left, growth curve of tumor volumes at indicated time. Middle, demonstration of tumor volumes of xenograft tumors removed from indicated group. Right, Statistical analysis of tumor weight of the resected tumors. Scale bars, 1 cm ^*^P < 0.05; ^**^P < 0.01. Student's-*t* test was used for the statistical analysis.

To determine the effects of Nrf2 on the biological behaviors of HCC cells, we first measured the proliferation activity of Hep3B and MHCC-97H cells by colony formation ability and Cell Counting Kit-8 (CCK-8) assay which allows sensitive colorimetric assays for the determination of cell viability in cell proliferation. Over-expression of Nrf2 in Hep3B cells significantly promotes cell growth and colony formation ability. Accordingly, ablation of Nrf2 in MHCC-97H cells showed decreased cell proliferation (Figure 2A and 2B). Consistently, Nrf2 depletion in Hep3B cells and forced expression of Nrf2 in MHCC-97H cells further verified this finding (Supplementary Figure S1A and S1B). In order to understand how Nrf2 regulates HCC cell growth, we tested the possibility that Nrf2 might affect cell cycle progression. To determine this, cell cycle analysis by PI staining was performed, which indicated that forced expression of Nrf2 displayed enhanced G1/S transition and cell cycle progression in Hep3B cells, while Nrf2 knockdown led to cell cycle arrest in MHCC-97H cells(Figure 2C). Additionally, Nrf2 ablation of Hep3B cells also promoted cell cycle arrest, while Nrf2 over-expression resulted in the opposite effect (Supplementary Figure S1C), suggesting Nrf2 boosted HCC cell growth by modulating cell cycle progression.

In a xenograft model, the tumors of Hep3B with Nrf2 overexpression had higher volumes and heavier weights than the control group (Figure 2D), and the tumors of MHCC-97H with Nrf2 inhibition had smaller volumes and lighter weights than the control group (Figure 2E).

Additionally, we generated a stable Nrf2 overexpression cell line of L02, which has low basic Nrf2 expression. The similar oncogenic effects induced by Nrf2 were observed both *in vitro* and *in vivo* (Supplementary Figure S2A–S2D). Taken together, these data suggest that Nrf2 promotes HCC cell proliferation both *in vitro* and *in vivo* that is associated with cell cycle progression of human HCC cell lines.

### Nrf2 possibly regulates cell cycle by activating the PDGFA/AKT pathway

**Figure 3:**
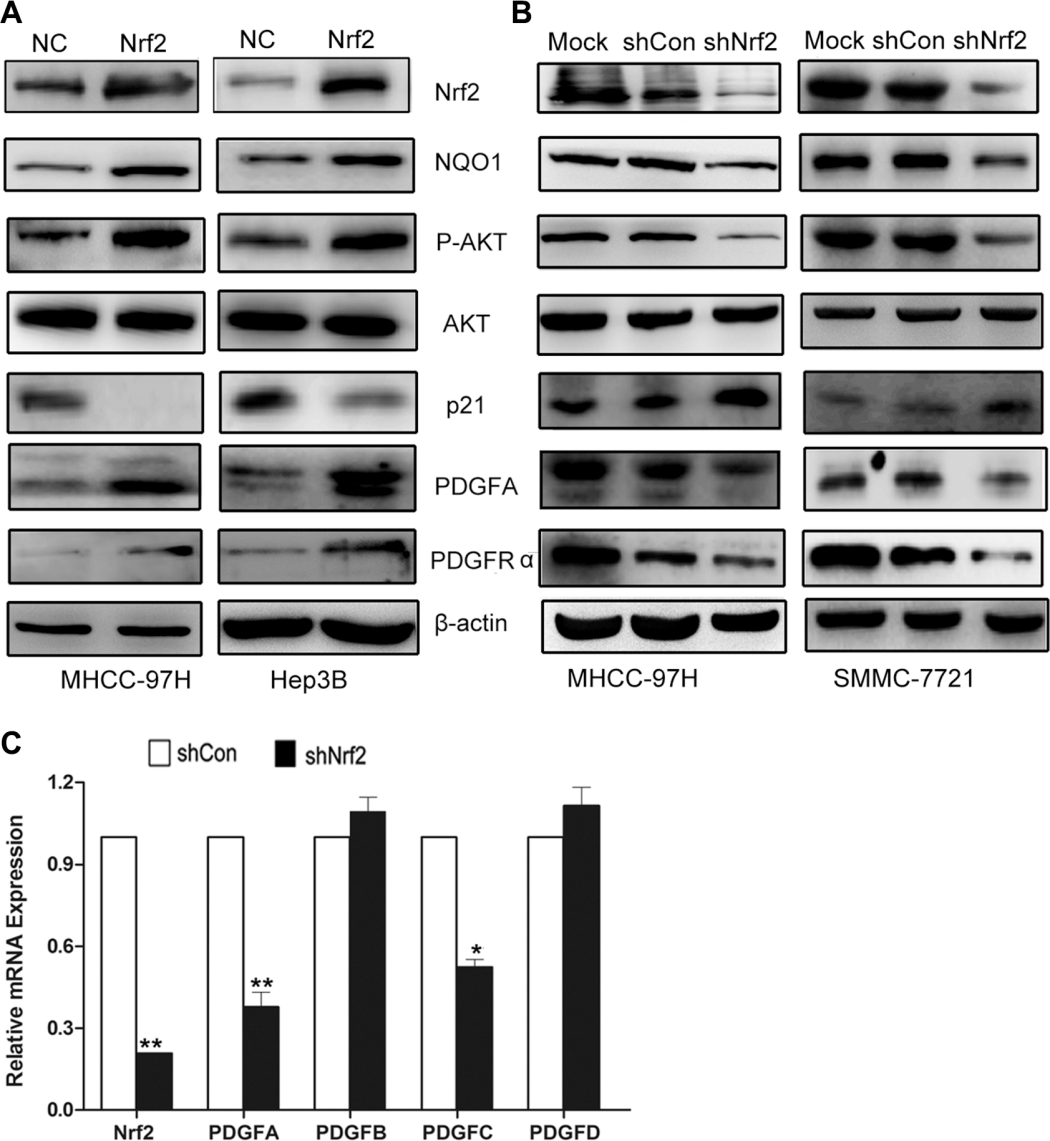
Nrf2 possibly modulates cell cycle progression by upregulating PDGFA and activation of AKT/p21 pathway (**A**) In Hep3B and MHCC-97H cells with Nrf2 overexpression, the protein expression levels of PDGFA, PDGFRα, phosphor-AKT (p-AKT) were significantly increased while the p21 expression decreased compared with the control. (**B**) Depletion of Nrf2 decreased PDGFA expression. The expression of indicated proteins was measured in MHCC-97H and MHCC-7721 cells by western blotting using indicated antibodies. (**C**) The PDGF family genes in MHCC-97H in response to Nrf2 stable knockdown were performed by Real-time PCR. Means ± SD from three independent experiments are presented. ^*^P < 0.05; ^**^P < 0.01. Student's-*t* test was used for the statistical analysis.

The AKT-dependent p21 pathway plays an important role in cell cycle progression [19, 20]. We therefore determined whether Nrf2 would modulate the cell cycle by controlling AKT/p21 signaling. As shown in Figure 3, Hep3B and MHCC-97H cells that overexpressed Nrf2, exhibited higher levels of AKT phosphorylation and decreased protein levels of p21 (Figure 3A, upper panel), as well as anti-oxidant-responsive element (ARE)-regulated gene including NQO1, whereas Nrf2 knockdown of MHCC-97H and SMMC-7721 cells significantly repressed the activation of AKT and increased protein levels of p21 (Figure 3B, upper panel). Further investigation showed that knockdown of p21 abrogated the tumor suppressive activity induced by Nrf2 knockdown in MHCC-97H cells (Supplementary Figure S3). These results suggested that Nrf2 activated the AKT/p21 pathway. It is well known that AKT activation is governed by multiple distinct mechanisms. Thus it would be interesting to figure out how Nrf2 regulates AKT/p21 pathway activation.

PDGFC, a member of Platelet-Derived Growth Factor (PDGF) family, was found as one of the inducible targets of Nrf2 by ChIP-PCR (Chromatin Immunoprecipitation PCR) assay [21]. We therefore doubted whether Nrf2 could regulate other PDGF family members. Interestingly, we found that PDGFA, but not PDGFB nor PDGFD, was significantly up-regulated by Nrf2, while PDGFC was modestly up-regulated (Figure 3C). Given that PDGFA was a well known activator of the AKT pathway [22] and was dramatically up-regulated in liver tumors [18], we postulated whether Nrf2 activated the AKT pathway by modulating PDGFA expression. Not surprisingly, we observed increased PDGFA protein levels in Hep3B and MHCC-97H cells with ectopic over-expressed Nrf2, while PDGFA was decreased in MHCC-97H and SMMC-7721 cells with Nrf2 knockdown, indicating Nrf2 was involved in regulating the proliferative activity of HCC cells by increasing PDGFA expression level (Figure 3A and 3B). Furthermore, the expression of its receptor PDGFRα was also induced by Nrf2 overexpression, possibly implying Nrf2 might be involved in positive feedback of PDGFA/PDGFRα signaling pathway (Figure 3A and 3B, lower panel). Collectively, these findings demonstrate Nrf2 promotes HCC progression by upregulation of PDGFA and possibly subsequent activation of the AKT pathway.

### Nrf2 interacts with and recruits Sp1 to PDGFA promoter for increased PDGFA expression

**Figure 4:**
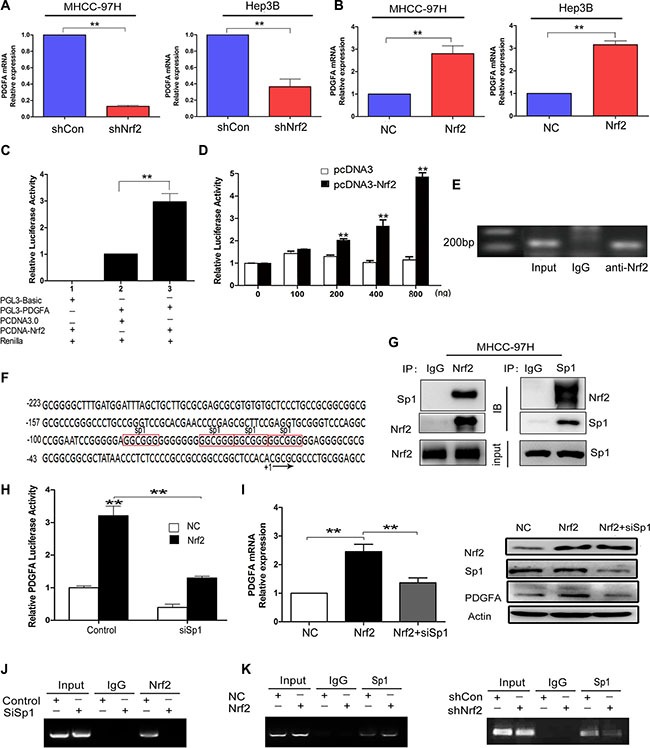
Nrf2 enhances the mRNA transcription of PDGFA by binding to PDGFA promoter via Sp1 (**A**) Ablation of Nrf2 downregulated PDGFA mRNA expression. MHCC-97H and Hep3B cells were infected with indicated lentivirus and the mRNA levels of PDGFA in indicated group were measured by qRT-PCR. (**B**) Ectopic expression of Nrf2 increased PDGFA mRNA expression. The mRNA levels of PDGFA in indicated group were measured by qRT-PCR. Means ± SD from three independent experiments are presented as relative ratio to the control whose value was taken as 1.0. (**C**) Nrf2 activated PDGFA promoter. MHCC-97H cells were co-transfected with indicated plasmids for 48 h. Means ± SD of normalized luciferase activity from three independent experiments are presented. (**D**) Nrf2 dose-dependently activated PDGFA promoter. HEK-293T cells cotransfected with PGL3-PDGFA, pRL-SV40, and various amounts (0, 100, 200, 400, 800 ng/well) of pcDNA-Nrf2 or pcDNA3.0 vectors. Means ± SD of normalized luciferase activity from three independent experiments are presented. (**E**) Nrf2 could bind to PDGFA promoter. ChIP assays were performed by immunoprecipitation chromatin fragments from MHCC-97H cells using anti-Nrf2 mAb or IgG control. 5% cell lysate was used as input. (**F**) Characterization of the region −223/+20 in PDGFA promoter revealed four binding sites for Sp1 (marked in red box). (**G**) Nrf2 interacts with Sp1 in the nucleus. Nucleoproteins were then extracted and subjected to Co-IP assay analysis. In this way we further revealed the interactions between Nrf2 and Sp1. 5% cell lysate was used as input. IB, immunoblot. IP, immunoprecipitation. (**H**) PDGFA luciferase activities were detected under the NC and Nrf2 overexpression group, respectively. Cells were transfected with no siRNAs (control), or Sp1 siRNA (siSp1). Means ± s.d. of normalized luciferase activity from three independent experiments are presented. (**I**) The upregulation of PDGFA by Nrf2 was dependent on Sp1. Cells that were infected with the indicated lentivirus were transfected with the indicated siSp1. The expression of PDGFA was measured by qRT-PCR and western blotting. (**J**) Sp1 is essential for Nrf2 binding to PDGFA promoter. ChIP assays were performed by immunoprecipitation chromatin fragments from MHCC-97H cells with Mock group or Sp1 depletion group using anti-Nrf2 mAb or IgG control. 5% cell lysate was used as input. (**K**) Nrf2 is required for Sp1 enrichment on PDGFA promoter. ChIP assays were performed with antibody against Sp1 or control IgG in MHCC-97H cells expressed high Nrf2 level or low Nrf2 level. Left, Nrf2 expression promotes Sp1 association with PDGFA promoter. Right, Nrf2 ablation attenuates Sp1 interaction with PDGFA promoter. ^*^P < 0.05; ^**^P < 0.01. Student's-*t* test was used for the statistical analysis.

Since PDGFA was identified as a putative target gene of Nrf2, we then verified whether PDGFA was regulated by Nrf2 transcriptionally. Indeed, PDGFA mRNA levels were significantly reduced after Nrf2 knockdown in MHCC-97H and Hep3B cells (Figure 4A), while they were significantly increased in MHCC-97H and Hep3B cells upon Nrf2 overexpression (Figure 4B). To analyze the ability of Nrf2 to regulate the PDGFA promoter, we co-transfected a luciferase reporter plasmid containing 1100 bp upstream of the PDGFA transcriptional start site into MHCC-97H cells along with pcDNA3.0 plasmid or Nrf2 plasmid (Figure 4C). With serial concentrations of the Nrf2 vector co-transfecting with Renilla control in HEK-293T cells, we demonstrated that Nrf2 could activate the transcription activity of PDGFA promoter dose-dependently (Figure 4D). Finally, ChIP assays suggested that immunoprecipitation by anti-Nrf2 mAb of chromatin fragments from MHCC-97H cells could specifically enrich PDGFA promoter sequences, indicating that Nrf2 is able to bind to the PDGFA promoter in HCC cells (Figure 4E).

Next we determined whether the binding of Nrf2 to the PDGFA promoter was direct or indirect. However, the typical Nrf2 binding motif (TGACnnnGC) was not found in the PDGFA promoter, implying that the binding is not direct. Previous studies demonstrated that Sp1 was the major transcription factor in controlling PDGFA expression (Figure 4F) [23, 24]. It is established that Sp1 can modulate DNA binding and transcriptional activity through an interaction with other proteins [25]. Nrf2 and Sp1 may form functional complexes that regulate HO-1 and transforming growth factor-β1 (TGF-β1) expression [26, 27]. We speculated whether Nrf2 triggered PDGFA mRNA expression by recruiting Sp1 similarly. To test the hypothesis, we first assessed protein interactions in nucleoprotein extracts. The immunoprecipitates enriched by Nrf2 or Sp1 antibodies were measured and we found Nrf2 indeed interacted with Sp1 (Figure 4G), which was consistent with previous studies. We then detected PDGFA transcription activity after transfection of Sp1 specific siRNA in control or Nrf2-overexpressed MHCC-97H cells. We found that Nrf2-overexpressed cells exhibited higher luciferase activity but significantly hindered its activity after silencing of Sp1 (Figure 4H). To further confirm the transcriptional dependence of Nrf2 *via* the Sp1 factor, we performed qRT-PCR and western blot assays and found that the mRNA and protein levels of PDGFA were reduced after transfecting Sp1 siRNA in Nrf2 over-expression cells (Figure 4I). In the ChIP assays, Sp1 was showed to be essential for Nrf2 binding to PDGFA promoter because Sp1 depletion abrogated Nrf2 enrichment on PDGFA promoter (Figure 4J). Moreover, ChIP assays revealed that the expression of Nrf2 significantly affected Sp1 binding to PDGFA promoter in Nrf2 over-expressed or Nrf2-silenced MHCC-97H cells (Figure 4K), indicating that, apart from Sp1, the constitutive level of Nrf2 in HCC cells was mandatory to manipulate PDGFA transcription. Taken together, our data here demonstrate that Nrf2 appeared to function as a coactivator and promoted PDGFA mRNA transcription *via* binding to and recruiting Sp1.

### Downregulation of PDGFA can partially abolish the proliferative activity of Nrf2

**Figure 5:**
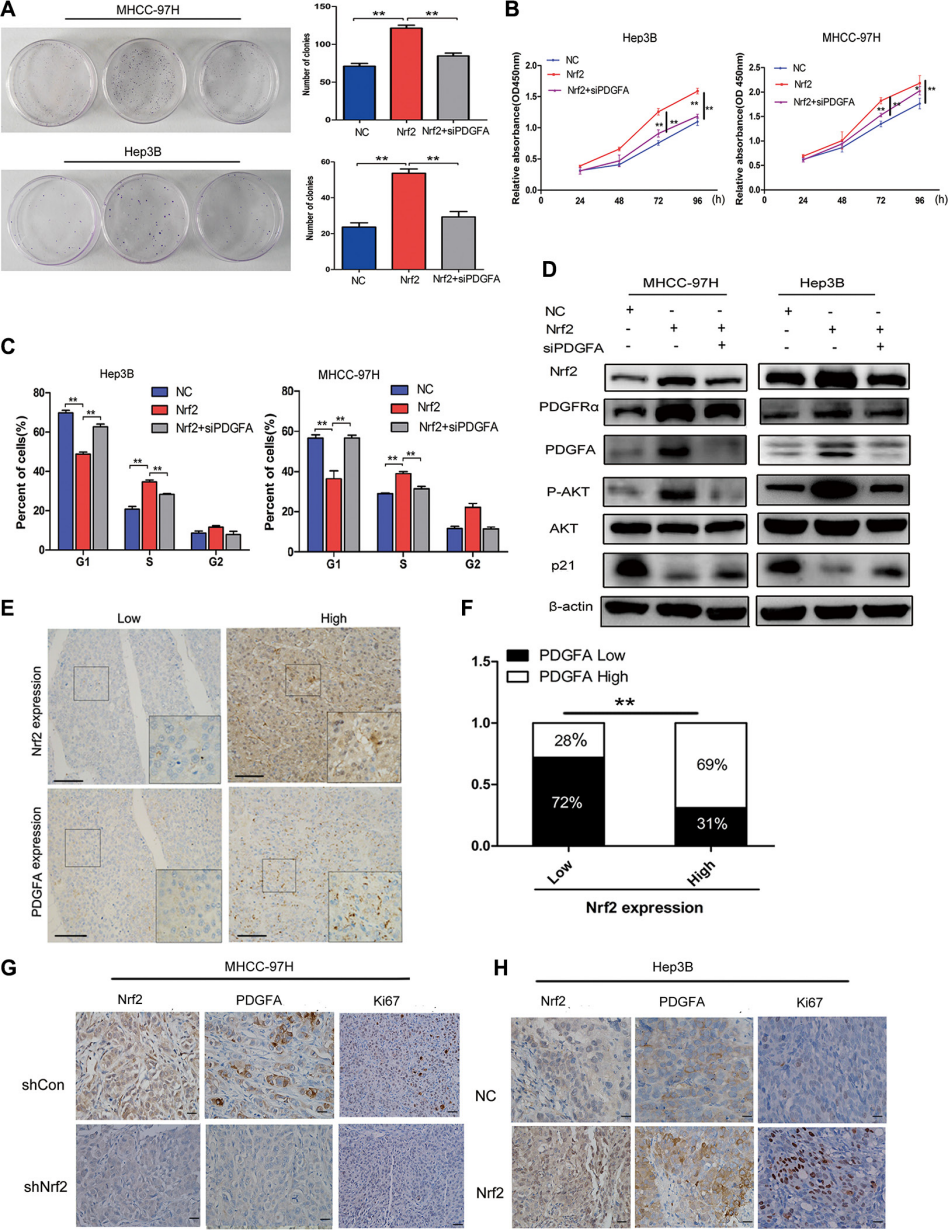
The tumor-promoting effect of Nrf2 is dependent on PDGFA (**A**) Colony formation (**B**) Growth curve (**C**) Cell cycle distribution. Depletion of PDGFA in Nrf2 over-expressed cells abolished the oncogenic activity of Nrf2. Cells infected with indicated lentivirus was transfected with siPDGFA. CCK8 assay, clony formation and cell cycle analysis were evaluated. (**D**) Depletion of PDGFA in Nrf2 expression cells reduced AKT/p21 pathway activation. Cells infected with indicated lentivirus was transfected with siPDGFA. The expression of protein levels were measured by western blotting using indicated antibodies. (**E**) IHC staining of Nrf2 and PDGFA. IHC results were independently graded by two experienced pathologists. Tissue samples with grades 0(no staining) and 1(weak staining) were grouped as ‘low’ expression, and samples with grade 2 (strong staining) were grouped as ‘high’ expression. Representative IHC staining images of Nrf2 and PDGFA are shown. (magnification ×400; Scale bars, 100 μm). (**F**) The expression of Nrf2 and PDGFA was positively correlated in HCC tissues. The correlation was analysed by Low-high bar graph using Graphpad software Prism 5. (**G**) and (**H**) Expression of Nrf2, PDGFA and Ki67 were positively correlated. Immunohistochemistry staining for indicated proteins in tumor tissues from mice with subcutaneous HCC implantation. (magnification ×400; ×100; scale bars, 50 μm). *P < 0.05; **P < 0.01. Student's-*t* test was used for the statistical analysis.

To gain a solid insight into the oncogenic role of Nrf2/PDGFA signaling in HCC progression, we sought to determine whether this role is dependent on PDGFA. We silenced the PDGFA expression in HCC cells that stably over-expressed Nrf2. CCK8 and cell colony formation assays showed that ablation of PDGFA blocked Nrf2 mediated proliferation of HCC cells (Figure 5A and 5B). Similarly, flow cytometry analysis showed that silencing of PDGFA blocked the activity of Nrf2 in promoting the entry of HCC cells in G1 phase into S phase (Figure 5C). In accordance with this phenomenon, down-regulation of PDGFA in Nrf2 over-expressed cells led to decreased AKT activation and increased p21 protein levels (Figure 5D).

Furthermore, we examined the expression and the correlation of Nrf2 and PDGFA in HCC tissues by immunohistochemistry analysis (IHC). In Nrf2 weakly staining samples, 72% showed low PDGFA expression. Similarly, 69% of Nrf2 strongly staining tissues were accompanied with higher level of PDGFA expression (Figure 5E and 5F), indicating Nrf2 and PDGFA expression are positively correlated in HCC tissues. We further found that patients with high expression of Nrf2 and PDGFA simultaneously displayed worse prognosis (Supplementary Figure S4). Results obtained from IHC analysis also demonstrated significantly lower protein levels of Nrf2 and PDGFA with less Ki67 positive staining cells in tissues from subcutaneous implantation models of MHCC-97H with Nrf2 inhibition compared with controls (Figure 5G) and Hep3B tumors with Nrf2 overexpression had higher PDGFA expression with more cells with Ki67 positive staining (Figure 5H).

### Nrf2/PDGFA feedback loop is critical for activation of the AKT pathway

**Figure 6:**
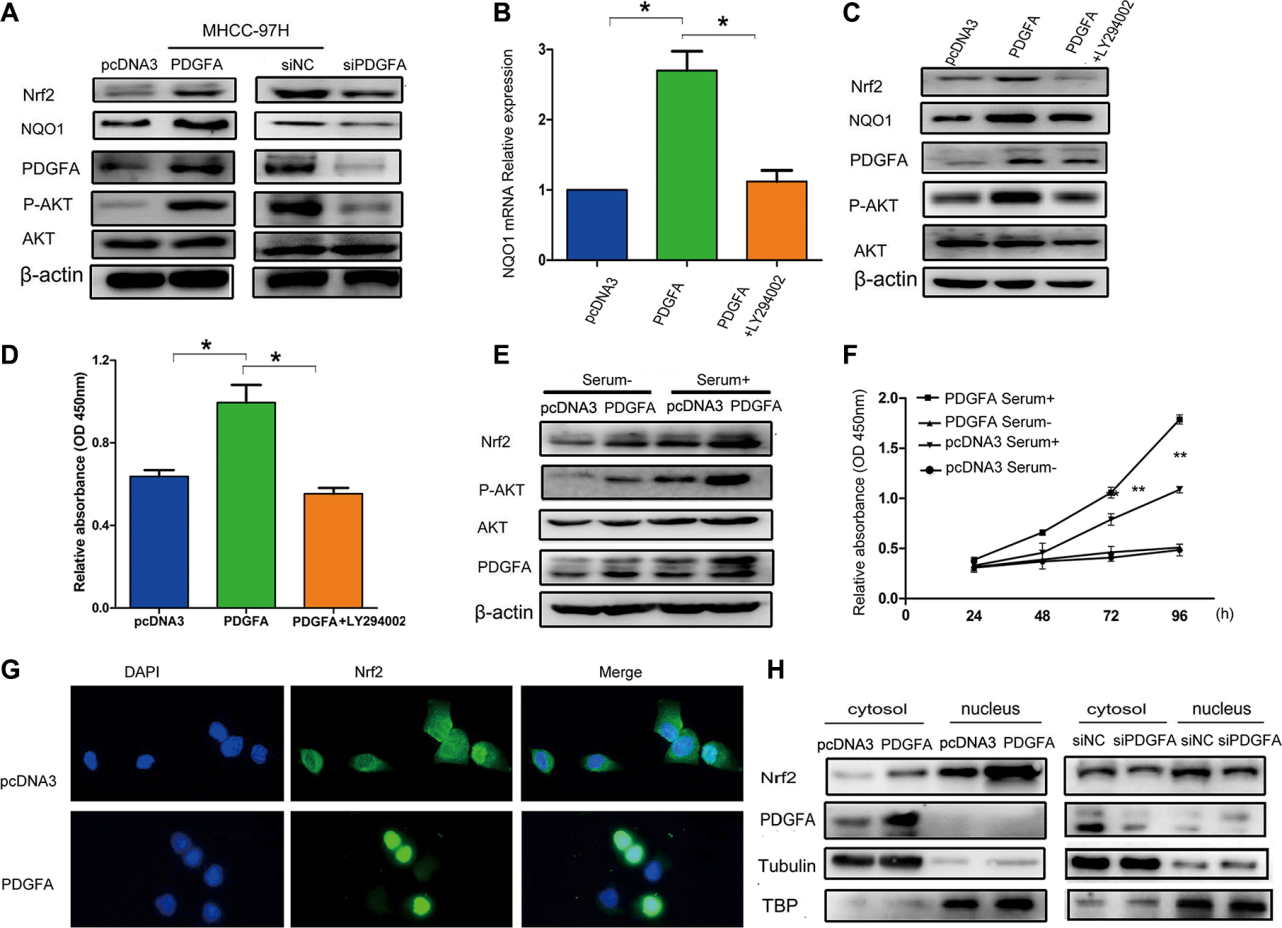
Nrf2 forms a feedback loop with PDGFA in the activation of the AKT pathway (**A**) Overexpression of PDGFA increased Nrf2 protein levels. MHCC97H cells infected with the indicated PDGFA plasmid and PDGFA siRNA were lysed and subjected to western blotting using indicated antibodies. (**B**) PDGFA up-regulated the mRNA expression of the Nrf2 downstream target gene NQO1 through the AKT pathway. MHCC97H cells infected with the indicated PDGFA plasmid and then treated with LY294002 (30 μM) for 24 hours were subjected to qRT-PCR using specific primers. (**C**) The role of PDGFA in promoting Nrf2 expression was dependent on the AKT pathway. Cells infected with the indicated plasmid was treated with LY294002 (30 μM) for 24 hours. The expression of Nrf2 was measured by western blotting using indicated antibodies. (**D**) MHCC-97H cell proliferation was analyzed by a CCK8 assay after cells were infected with PDGFA or PDGFA plus LY294002. (**E**) Active AKT signaling in cultured cells enhanced PDGFA-mediated modulation of Nrf2. MHCC-97H cells infected with indicated plasmids were maintained in cell culture medium containing serum or serum-free medium, followed by western blotting analysis of Nrf2 expression using the indicated antibody. (**F**) AKT activation enhanced PDGFA-mediated cell survival by Nrf2 upregulation. MHCC-97H cells infected with indicated plasmids were maintained in cell culture medium containing serum or serum-free medium. Cell viability was assessed by CCK8 assays. Means ± SD of relative absorbance from three independent experiments are presented. (**G**) Immunofluorescence revealed that exogenous PDGFA expression promoted Nrf2 translocation to the nucleus in MHCC-97H cells. (**H**) Cell fractionation assays revealed that PDGFA increased the nuclear Nrf2 protein levels in MHCC-97H cells. Tubulin and TATA box binding protein (TBP) were used as loading control.^*^P < 0.05; ^**^P < 0.01. Student's-*t* test was used for the statistical analysis.

Previous studies and our current work revealed that Nrf2 and PDGFA are both able to activate the AKT pathway [28, 29]. This leads us to speculate whether Nrf2 and PDGFA form a feedback loop for coordination of AKT activation. To test this hypothesis, the protein levels of Nrf2 were detected in the MHCC-97H cells that were infected with PDGFA or PDGFA siRNA, which indicated that forced expression of PDGFA induced elevated Nrf2 expression and increased AKT activation, while depletion of PDGFA reduced Nrf2 protein level and attenuated AKT phosphorylation (Figure 6A).

Inhibition of the PI3K/AKT pathway can reduce nuclear retention of Nrf2 and its ARE binding affinity [30]. Therefore, we determined whether PDGFA regulated Nrf2 through the AKT pathway. We observed that PDGFA activated NQO1 transcription, which is the downstream target gene of Nrf2 and this activation was blocked by LY294002, an inhibitor of the AKT pathway (Figure 6B). Furthermore, inactivation of AKT by its inhibitor LY294002 in PDGFA expression cells abolished Nrf2 upregulation, at least partially, suggesting AKT activation was indispensable for the elevation of Nrf2 by PDGFA (Figure 6C). Also, LY294002 blocked cell growth induced by PDGFA (Figure 6D). PI3K-Akt signaling could be turned off by serum starvation, and we found that the activation of AKT was dependent on the presence of serum. Then AKT activation led to PDGFA to trigger a higher level of Nrf2 compared with cells that underwent serum deprivation, resulting in increased HCC cell survival (Figure 6E and 6F). The data indicated that active AKT signaling in cultured cells could enhance PDGFA-mediated modulation of Nrf2.

Furthermore, by using immunofluorescence staining and cell fractionation assays, we found that upregulation of PDGFA in MHCC-97H cells led to enhanced Nrf2 nuclear translocation (Figure 6G and 6H). Collectively, these data here describe that the Nrf2/PDGFA feedback loop through AKT activation is critical for HCC progression.

## DISCUSSION

Nrf2 has been regarded as a tumorigenic factor in various types of tumors by inhibiting cellular apoptosis, increasing drug resistance and promoting oncogenesis [7, 31]. Several studies have identified that mutations of Nrf2 are extremely frequent in rat and human HCCs. These mutations lead to tumor initiation and malignant transformation in liver carcinogenesis [10, 32]. However, the mechanisms of Nrf2 induced HCC progression are poorly understood. Our study comprehensively showed that both mRNA and protein levels of Nrf2 were remarkably upregulated in clinical HCC specimens and correlated with poor prognosis. We presented, for the first time, that Nrf2 promoted HCC proliferation by activating PDGFA and forming a forward feedback loop with the PDGFA/AKT signaling pathway.

Accumulating evidences have established that excessive proliferation is one of the fundamental hallmarks of cancer cells through deregulated cell cycle progression and resistance to cell death [33]. It has been shown that Nrf2 promotes HCC cell survival by suppression of apoptosis via upregulation of BCL2L1, a potent inhibitor of cell death that represses activation of caspases [34]. Hypothesizing that Nrf2 might play multiple roles in HCC progression, we attempted to determine whether Nrf2 could regulate cell cycle control of HCC cells and we observed that Nrf2 indeed promoted G1/S phase transition, resulting in increased cell cycle progression (Figure 2C). In line with these observations, we found that Nrf2 could promote tumor growth *in vivo* in a xenograft mouse model (Figure 2D and 2E). Taken together, these results suggest that Nrf2 is indeed a positive regulator of HCC cell growth both *in vitro* and *in vivo.*

Cell cycle progression is controlled by the activity of cyclin-dependent kinases (CDKs) and its inhibitors (CKIs) including p21^Waf/Cip1^, which is one of the chief substrates of AKT, a critical regulator of cell proliferation and survival [35, 36]. Accordingly to our findings, AKT can be activated by Nrf2, leading to the degradation of p21, suggesting Nrf2 promoted HCC progression by activation of the AKT/p21 pathway. Next we determined how AKT/p21 signaling was regulated by Nrf2. Previous literature reported that PDGFC was a target gene of Nrf2 in one ChIP-Seq profiling experiment [21]. We therefore confirmed whether the PDGF family (PDGFA, B and C) could be regulated by Nrf2. Interestingly, PDGFA could be transactivated by Nrf2. Previous studies have shown that the autocrine PDGFA/PDGFRα signaling pathway can activate the PI3K/AKT pathway and promote HCC progression [17]. Inhibiting PDGFRα expression or activity by reducing its phosphorylation is an effective strategy for molecular therapy in HCC or other tumors [18]. Our study indicated that Nrf2 expression in HCC could not only increase PDGFA generation but also upregulate PDGFRα expression, leading to autocrine signaling in tumor cells (Figure 3A and 3B). In line with this, we demonstrated that the function of Nrf2 was dependent on PDGFA as evidenced by decreased oncogenic activity upon PDGFA depletion in Nrf2 expression cells (Figure 5A–5D). Furthermore, in HCC tissue samples, Nrf2 and PDGFA expression were found to be positively correlated (Figure 5E).

ChIP assays indicated that Nrf2 was able to bind to the PDGFA promoter (Figure 4E). However, the specific binding motif of Nrf2 is not found in PDGFA promoter, suggesting Nrf2 indirectly associates with the promoter. Several studies demonstrated that Nrf2 could interact with Sp1 and c-Jun for binding and transcriptionally regulated some downstream genes [26, 27]. Sp1 is also a well-known positive regulator of PDGFA transcription [23]. In agreement with these findings, we demonstrated that Nrf2 interacted with Sp1 in the nucleus and recruited Sp1 to PDGFA promoter for increased PDGFA transcription (Figure 4G). Additionally, transcriptional activation of PDGFA by Nrf2 was Sp1-dependent. After interaction with Sp1, Nrf2 functioned as an activator of PDGFA, whereas knockdown of Sp1 eliminated the increased effect of Nrf2 on PDGFA transcription (Figure 4H–4J). The data highlights, rather than the direct binding to ARE sequence, Nrf2 could coordinate with Sp1 for selective PDGFA expression but not PDGFB and PDGFD.

The tumor positive feedback pathway is a common mechanism to amplify a response and thereby activate factors or signaling pathways that are autonomous to the original stimuli in tumor progression. In our study, we found Nrf2 activated the AKT pathway through PDGF-A. The PI3K/AKT signaling pathway is a major contributor to cancer development. Studies have shown that inhibiting PI3K/AKT activity reduced the nuclear accumulation of Nrf2 and increased Nrf2 ubiquitination with a concurrent decline in its downstream targets [30, 37]. In line with this, we also proved that PDGFA could increase Nrf2 expression and transcriptional activation in an AKT-dependent manner in HCC cells (Figure 6A–6C). It is tempting to speculate that the Nrf2/PDGFA feedback loop allows HCC cells to become more autonomous. These findings verified that the activation of AKT by the Nrf2/PDGFA feedback loop was critical for HCC cells growth and survival.

Furthermore, we observed that PDGFA could promote Nrf2 translocation to the nucleus in HCC cells (Figure 6G and 6H). This phenomenon can be explained by two possible mechanisms. First, PDGFA can increase Nrf2 expression and accumulation in the nucleus by persistently activating its downstream PI3K-AKT signaling pathway [38]. There is evidence in the literature that GSK-3 can promote the KEAP1-independent degradation of Nrf2 [39]. Because AKT phosphorylates GSK-3 and inhibits its activity [40], the active PI3K-AKT signaling should stabilize Nrf2 by suppressing GSK-3. Second, PDGF can activate the small GTPase Rac1 that is important for the activation of NAD(P)H oxidase, a major source of ROS [41, 42]. Nrf2 translocates to the nucleus in response to ROS, thereby inducing related genes to increase malignancy and chemoresistance [43].

In summary, our findings show the link between Nrf2 and the PDGF/AKT signaling pathway in human hepatocellular carcinoma. We firstly discover that Nrf2 transcriptionally activates PDGFA expression *via* interacting with Sp1. Our current study describes a novel mechanism for AKT signaling involving Nrf2 and PDGFA, which might provide prognostic value and possible therapeutic targets for HCC.

## MATERIALS AND METHODS

### Patient samples

A total of 79 patients were enrolled in the study. Human HCC and their paired non-tumorous liver tissues were collected during surgical resection at Huashan Hospital, Fudan University between January 2009 and December 2011. The samples were snap-frozen in liquid nitrogen and stored at –80°C for later RNA extraction or formalin-fixed and paraffin embedded for imunohistochemistry. Patients were followed after surgical treatment until May 2015, with a median follow-up of 50 months. Histopathology was evaluated by two certified pathologists in the department of pathology at Huashan Hospital of Fudan University. All human materials were obtained with informed consent and approved by the Ethics Committee of Huashan Hospital of Fudan University.

### Reagents, plasmids and siRNA

Antibodies used in western blot include anti-Nrf2 (ab137550, Abcam, Cambridge, UK), anti-PDGFA (sc-128, Santa Cruz, USA), anti-PDGFRα (sc-338, Cell Signaling Technology, USA), anti-phosphor-AKT (Ser473) (#9271, Cell Signaling Technology, USA), anti-AKT (#9272, Cell Signaling Technology, USA), anti-NQO1 (sc-271116, Santa Cruz, USA), anti-p21 (#2947, Cell Signaling Technology, USA) and anti-Sp1 ( #5931, Cell Signaling Technology, USA). The mouse anti-β-actin (A1978, Sigma, USA) monoclonal antibody was used as a sample loading control. The phosphatidylinositol 3-kinase (PI3K) inhibitor LY294002 was purchased from Cell Signaling Technology. Dual-Luciferase Reporter Assay System was purchased from Promega (Madison, WI, USA). The 1100-bp fragment of human PDGFA promoter was generated by PCR amplification from Genomic DNA of human blood and subcloned into PGL3-basic luciferase reporter plasmid (Promega). Nrf2 expression plasmid is a gift from Dr. Donna Zhang from the Department of Pharmacology and Toxicology, University of Arizona. PDGFA cDNA was cloned in pcDNA3 (Invitrogen, Carlsbad, USA). For knockdown of PDGFA and Sp1, specific siRNAs were designed and chemically synthesized by GenePharm Co.Ltd (Shanghai, China). The sequences for PDGFA siRNA, SP1 siRNA and p21 siRNA were as follows: 5′-CUGAAUCCGGAUUAUCGGG AA-3′; 5′-GACAGGUCAGUUGGCAGACUCUACA-3′; 5′-AAUGGCGGGCUGCAUCCAGGA-3′ respectively. Transfection of siRNAs or plasmids was performed using Lipofectamine 2000 (Life Technologies) regent according to the manufacturer's instructions.

### Cell culture and lentivirus preparation

The human HCC cell lines PLC, Hep3B, SMMC-7721, Huh7, HepG2, the normal liver cell line L02 and embryonic kidney cell line HEK-293T were purchased from the Cell Bank of Typical Culture Preservation Committee of Chinese Academy of Science (Shanghai, China). Human HCC cell lines MHCC-97H and HCC-LM3 were established at the Liver Cancer Institute, Zhongshan Hospital, Fudan University. These cells were stored in liquid nitrogen and cultured in 5% CO2 at 37°C with high glucose Dulbecco's modified Eagle media (GIBCO, Grand Island, NY) supplemented with 10% FBS (GIBCO).

The negative control small interference RNA (siNC, 5′-UUCUCCGAACGUGUCACGUTT-3′), and Nrf2 siRNA (5′-CAUUGAUGUUUCUGAUCUATT-3′) were synthesized and inserted into the LV3-GFP vector (named as LV3-shCon or LV3-shNrf2). For Nrf2 overexpression, the Nrf2 expression plasmid was cloned into LV5-GFP vector (named as LV5-Nrf2). Thereafter, HEK-293T cells (1 × 10^6^ cells) were transfected with the respective plasmid (LV3-shCon, or LV3-shNrf2, or LV5-NC, or LV5-Nrf2) and packaging vectors by GenePharma Co. Ltd (Shanghai,China). HCC cells were then infected with the packaged lentivirus under medium culture.

### RNA isolation and quantitative real-time PCR (qRT- PCR)

Total RNA was extracted from cell lines and frozen tumor specimens using Trizol reagent (Invitrogen, California, USA). Quantitative PCR was performed using qPCR Master Mix for SYBR Green (Takara, Shiga, Japan) and the ABI7900Fast Sequence Detection system. The thermal cycle condition was one cycle at 95°C for 30 s, followed by 40 cycles of amplification at 95°C for 5 s, and then 60°C for 30 s. All samples were run in triplicate in each experiment. Values were normalized to that for GAPDH. The sequences of the primers used were showed as follows:

hNrf2-f/hNrf2-r: ACACGGTCCACAGCTCATC/TGTCAATCAAATCCATGTCCTG; hNQO1-f/hNQO1-r: ATGTATGACAAAGGACCCTTCC/TCCCTTGCAGAG AGTACATGG;

hPDGFA-f/hPDGFA-r: ACTAAGCATGTGCCCGA GAA/GTAAATGACCGTCCTGGTCTTG; hPDGFB-f/hPDGFB-r: CTCGATCCGCTCCTTTGATGA/CGTTGGT GCGGTCTATGAG

hPDGFC-/hPDGFC-r: GACTCAGGCGGAATCCA ACC/CTTGGGCTGTGAATACTTCCATT

hPDGFD-f/hPDGFD-r: TTGTACCGAAGAGATGAGACCA/GCTGTATCCGTG TATTCTCCTGA

hSp1-f/hSp1-r: AGTTCCAGACCGTTGATGGG/ GTTTGCACCTGGTATGATCTGT hGAPDH-f/hGAPD H-r: CTGACTTCAACAGCGACACC/TGCTGTAGCC AAATTCGTTGT.

### Chromatin immunoprecipitation (ChIP)

ChIP assay was performed using the EZ-chip kit (17–371, Millipore, USA) according to the manufacturer's instructions. The cross-linked protein- DNA complexes were immunoprecipitated using an anti-Nrf2 antibody (Abcam, Cambridge, UK) and Sp1 antibody (#5931, Cell Signaling Technology). Precipitated DNAs were analyzed by PCR. Primers were designed to amplify a 220 bp region in human PDGFA promoter (−243 to −20) containing the sp1 site and the sequences were as follows: 5′-GCCCCGGCGCGGAGCCGGC-3′ and 5′-GCGGGC TCCGCAGGCGCGC -3′.

To detect the interactions of endogenous proteins, nucleoproteins were first extracted and subsequently immunoprecipitated with 5 μg of primary Ab for 10 h followed by incubation with Protein A–agarose beads (sigma, Germany) for 1 h. The precipitates were then eluted by boiling in 1 × SDS loading buffer for western blotting analysis.

### *In vitro* cell proliferation, cell cycle, and colony formation

The proliferation of HCC cells *in vitro* was measured using the Cell Counting Kit-8 (DOJINDO, Kumamoto, Japan). 3000 stably infected cells were seeded into each well of 96-well plate (*n* = 6 for each time point) in a final volume of 100 μl. Then CCK-8 solution (10 μl) was added into each well, and the absorbance at 450 nm was measured after incubation for 2 hours at 37°C to calculate the number of viable cells.

For cell cycle analysis, 1 × 10^6^ cells were harvested and washed in PBS, then fixed in 75% alcohol for 60 min at 4°C. Cell cycle analysis was prepared with a cell cycle detection kit (Keygen biotech, Nanjing, China). The cell cycle was analyzed by flow cytometry.

For colony formation assays, 500 cells were seeded into 35 mm dishes (Corning Costar Corp, Corning, NY). Then the cells were incubated at 37°C in a humidified atmosphere containing 5% CO_2_ in air for 2 weeks. Subsequently, we removed the medium and stained the cells with crystal violet (Beyotime Institute of Biotechnology, Beijing, China), captured the dishes with a camera (Nikon, Tokyo, Japan). Only positive colonies (diameter > 40 um) in the dishes were counted and compared.

### Immunohistochemical staining

The paraffin-embedded tissues were sectioned at 5-μm thickness, deparaffinized in xylene, rehydrated in graded ethanol solution and immersed in 0.1 M citric acid buffer (PH 6.0) for 12 min at 115°C, 121 kPa. Slides were incubated with the primary antibodies against human PDGFA (1:50; sc-128, Santa Cruz, USA), Nrf2 (1:100; ab137550, Abcam, Cambridge, UK) and Ki67 (1:100; sc-23900, Santa Cruz, USA) overnight at 4°C. The sections were washed with phosphate-buffered saline and subsequently colored using the Dako Envision system/HRP (Dako Cytomation, Denmark).

### Immunofluorescence

Cells were grown on glass coverslips, then transfected with PDGFA plasmid. 24 h later the cells were fixed and incubated with anti-Nrf2 (1:100, ab137550, Abcam, Cambridge, UK) followed by incubation with anti-rabbit secondary antibody conjugated to Alexa-488 (Invitrogen Molecular Probes). Nuclei were stained with DAPI (Sigma). Intracellular localization was determined by fluorescence microscopy (Vectra, USA).

### Xenograft studies

Male BALB/c nude mice at 4 weeks of age were obtained from SLRC (Shanghai, China) and maintained in pathogen-free conditions in accordance with the National Institutes of Health Guide for the Care and Use of Laboratory Animals. To determine the role of Nrf2 on HCC growth *in vivo*, 2–4 × 10^6^ cells of stable cell lines MHCC-97H, Hep3B and L02 suspended in 100 μl of PBS were implanted into the flank of nude mice. Tumor size was measured every 4 d for 20 to 30 d, and tumor growth was quantified by measuring the tumors in three dimensions with calipers. Tumor volumes were calculated using the following formula: tumor size (mm^3^) = width^2^ × length × 0.52. At the end of the experiment, all the animals were euthanized, and tumors were excised and weighed.

### Statistical analysis

Statistical analyses of the data were performed using STDEV and the *t* test with the SPSS 13.0 (SPSS Inc, Chicago, IL) statistical software package and GraphPad Prism 5.0 (GraphPad, San Diego, CA, USA). Probability values of less than 0.05 were considered significant.

## SUPPLEMENTARY MATERIALS FIGURES


