# Supplementary material for: Activation of AKT pathway by Nrf2/PDGFA feedback loop contributes to HCC progression
Source: Oncotarget. 2016 Aug 30;7(40):65389–402. doi: 10.18632/oncotarget.11700 (PMC5323163; doi:10.18632/oncotarget.11700)
Supplement: Supplementary file 1 [file oncotarget-07-65389-s001.pdf]

Activation of AKT pathway by Nrf2/PDGFA feedback loop contributes to HCC progression

Supplementary Materials

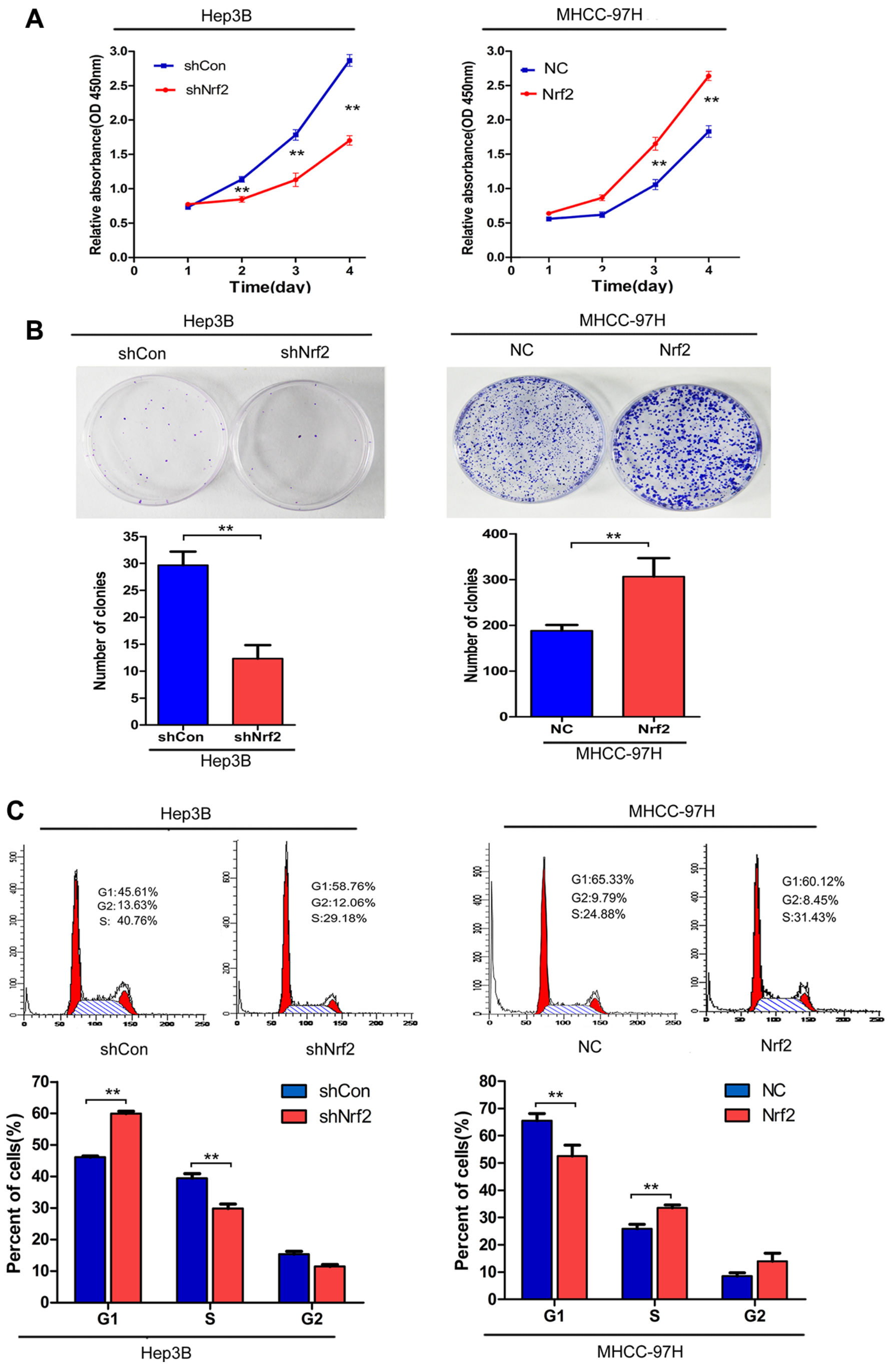

Supplementary Figure S1: Nrf2 enhances HCC cell proliferation in Hep3B and MHCC-97H cells. (**A**) Nrf2 affected cell growth in Hep3B and MHCC-97H cells. Equal numbers of the cells infected with indicated lentivirus were plated into 96-well plates. The proliferation activity was measured by CCK8 assay. (**B**) Nrf2 triggered the colony formation ability. 500–1000 cells that infected with indicated lentivirus was plated into 35 cm dish for 2 weeks. The numbers of colonies was counted and calculated. (**C**) Nrf2 governed HCC cell cycle progression. 10^6 cells that infected with indicated lentivirus were analyzed by FACS for the relative percentages of cell cycle phases. Representatives are results of at least three independent experiments in triplicates. $^*P < 0.05$; $^{**}P < 0.01$. Student's-*t* test was used for the statistical analysis.

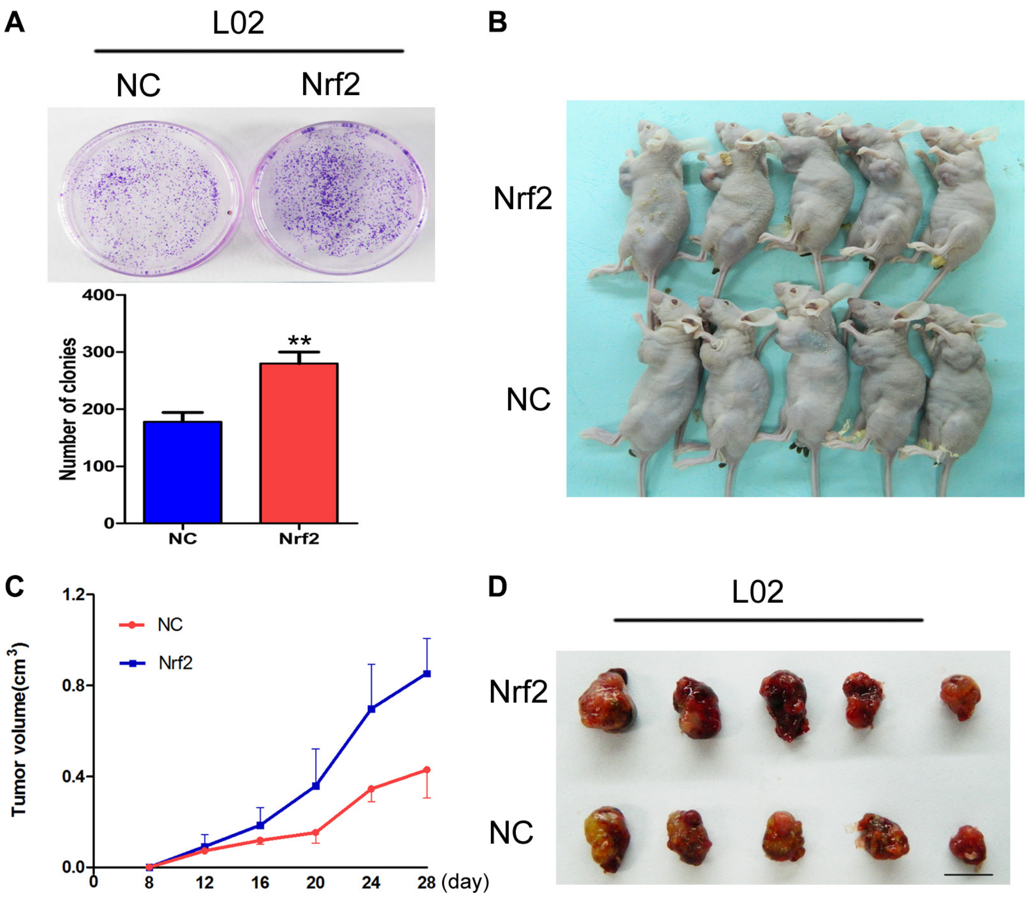

Supplementary Figure S2: Nrf2 enhances L02 cells proliferation *in vitro* and *in vivo*. (**A**) Colony formation. Nrf2 triggered cell growth in L02 cells. Equal numbers of the cells infected with indicated lentivirus were plated into 35 cm dish for 2 weeks. The number of colonies was counted and calculated. (**B**) Effect of Nrf2 on growth of L02 xenograft in nude mice. Xenografts were established by subcutaneous injection of indicated clones to the nude mice. Five mice were used in each group ($n = 5$). (**C**) Growth curve indicated the tumor volume of xenografts at the indicated days after injection. (**D**) Tumor volumes in the orthotopic implantation models at week 4 were shown. Scale bars, 1 cm. *$P < 0.05$; **$P < 0.01$. Student's-*t* test was used for the statistical analysis.

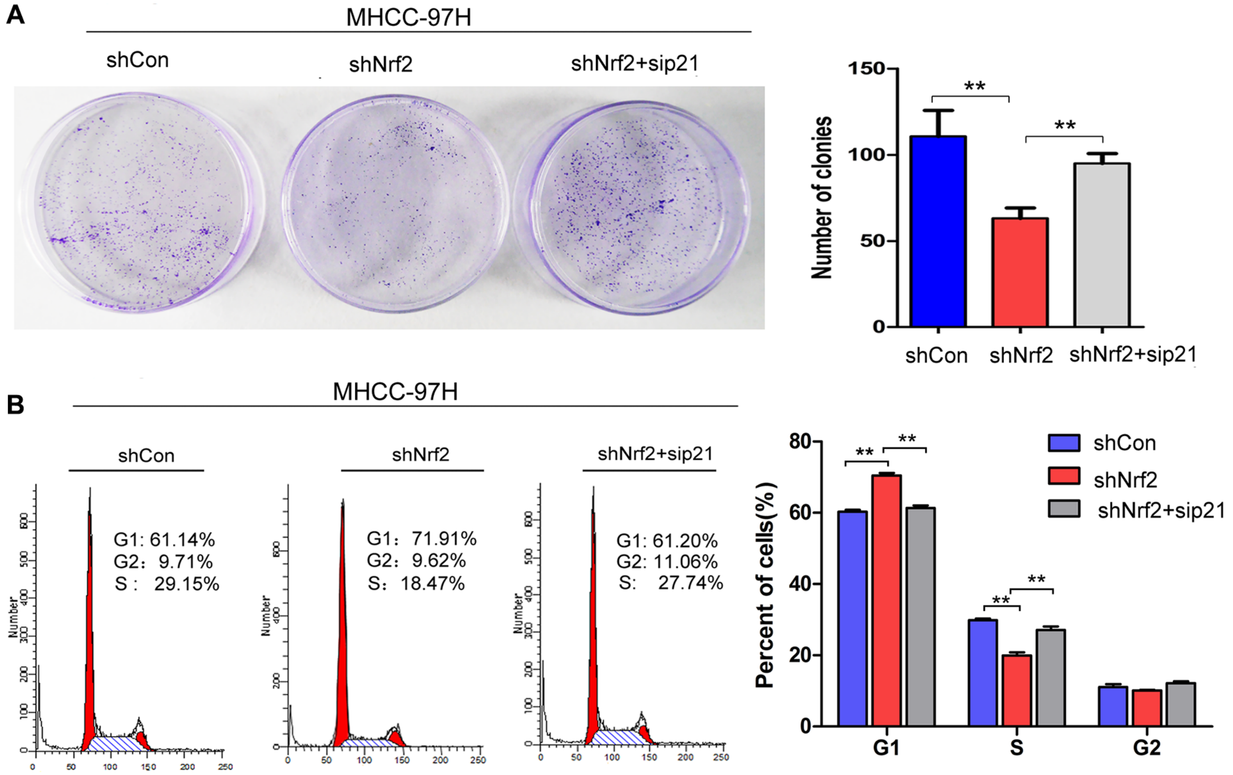

Supplementary Figure S3: Nrf2 promotes tumorigensis in a p21 dependent manner. (**A**) Colony formation. (**B**) Cell cycle analysis. MHCC-97H cells infected with indicated lentivirus were analyzed using colony formation and FACS-based cell cycle calculation. Representatives are results of at least three independent experiments in triplicates. $^{*}P < 0.05$; $^{**}P < 0.01$. Student's-*t* test was used for the statistical analysis.

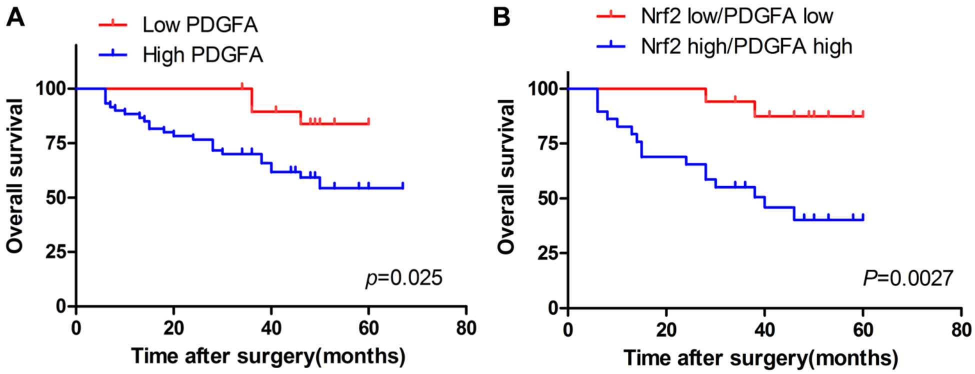

Supplementary Figure S4: The prognostic significance of Nrf2 and PDGFA for HCC patients was assessed *via* Kaplan-Meier analysis. The patients with higher PDGFA had (**A**) poorer overall survival (OS). (**B**) Patients in subgroup low Nrf2/low PDGFA had longer OS than subgroup high Nrf2/high PDGFA. The combination of high Nrf2 and high PDGFA expression increased the probability of poor prognosis.
